# Record of Meteorological Conditions at Buffalo, for April, 1855

**Published:** 1855-06

**Authors:** Sanford B. Hunt


					﻿ART. VI.— Record of Meteorological Conditions at Buffalo, for April,
1855. By Sanford B. Hunt, M. D.
tn
»
cj
a.iruBjadiUrfj, [	©
jo aitinM jsojnojjjjx©©©ci©cit-t^©©t'.©noct©© — ct©©©©Cl©©©^	©’
An - © © .o---<- © _ >r. — jo ci © qi t- j_ ~ — c~. _ © x x '—	©
ntrnnti irnjr.- 7©2jD(;Cl-© cirtMO-ioc© ©c-tcco<cototo©cci	©
p.llinil 11'. ||X	, I~ f <-	7	7	/	7	/	— _ — I- X — I — I- l~ — <-©<-©	<-
u	I — ©	©x	© © ©	.^<ci©©r-x	—	©t-©xm©©©	©
<	M9(J UBJt5	1 r4 ©	-4	X	71 O CO	© © Ci	zi	71	t— © 71	(4 — -4 71	CO	© zi Ci	CO © © Ct —	©
j 3 ______I—■ — CtCt©©Ti©©©©©©7j<©©Tj«©©-^<©©7t<Tr,0,TI'©©_________________________________________________©
•ainju	i co co	.	t'-	. .coco .cococo	.co	© ©	©	t—	.no	© . ©©	|©
-Jadtnaj. ub >i\', -r ©’	r4	©	no r4 co	© —2 t—	~	~	co’ © no	x —: 36	<4	ci x ci	x © — co to
______CQ	CQ	co	~? r*7 co	tj* -?< co	co	~r	-y t t	~ 10 .o v-O	io uo	»-O »o cc ry tt1	yy
-	t c: x - c:	r. —»	o	— — ao	— o c* o	i”3>
•Xiinimnu ®	c r:	x 75 O	Q? O CO 30	|uO
I. AJ^-	'sC XCC-'^ XX'JOXX^O'XCO GO O X H	C-
Q>______I___________________________ —___________________________________________________________________I
E -lIIKijr MAi-r 1.05 S>©CTCl©n>©Cl©©Cl©t-	©	O
•	n _____I——i — Cl © ct ct ©© ct ©©©-c ©-7 oocom__________________________________________________________co
X	. I	©
X,	•	''',-l'. lo-r l'. n ?> c ci--, nee i-co x no cocc-t’	x
________odciWTcon-concccoo-fcoo © -n* -q* -»_____________________________________________________________©
OT 'O	••	•	•
x ,o j S ® f	02	02 02 02	02 02
=	5 ?	. .	...	.....
. f- ________-1* — O©©©— — JOOOO — — —	—' — O O________________________________________________
Z2 spiio|Q jo i.iu v |	—> — o©©o©©o©o-t©oo © © © ct
o;	0 00 33 c no>4 ©©©©—© ofTo ©'©_—J x © t— © t— r- © © -7 71 ©	© i©
g	.	"‘©©©ClnOCl — <'-7©©©Clr-t-©—©<^© > I- no t-f -7 X X © cl
7	AJipiUItl JJ © ©X © © X © t- t— X t- © X © L-; if. t- H X X T © 1.0 7 (' C © -r©X 11—
E 1UIOJM.W1 . t^OTP . 00 . nO nO-7 © 71 — X © © © Cl-7 t- X © Cl X . ©Cl . co-0J	let
. n _____Ci — c ©© © ci © © © ©	" 7 >-. ic © -t © © - © — — © © © ©	'©
*	I i?	|
, • 9jn2.duiax © cj © © © ci o -7 ©’ ci ©‘ © © x’ o t4 — -4 —' o' -o< o cf •* © © ©’ ci o o ©'
— _________d©©-r©~r-©'©©©c?—r-7no©©©©©©©©©©©7t<©©©
• . . £bB=	• •
£5c=2	02 02	02 02 02'^	02 02	02 02’X 02 02	02 02 02	02
......................................................
______________________________________________________©©©©— ©©Cl©Cl©ClC7'-'—|©C>© — ©r-.Clr-i’-i—©©Cl «__
|spno[f)joj.mv coo©* ©©©©©©©co©©©©©ooco©CX©o©©
| © .O © © © © © © — — X X © © © X Ct © © © Cl ©	© © — —	I©
U ■a"‘!P!luuH |(^ ©C0XXXXX©©XXX©©©C-X©XXX©Xt~-©t~t-	[X
E	uo/. 75 t~ .Cl© Cl CICIX	©©©© — ©—. Cl no X©	?l
•	o 5U! >d M;’(l _ ,70 O ©<' — © — 'O no ©©—’©’© ci x' © © © o — C --- <- Ci Ct	©
•=1 St____Cl — Cl Cl Cl © Cl ©©© Cl ©©©©©© no ©©©©©©©© — 70________________________________________________CO
*	2s*
ajnj.diuax co co x © [4. x t4 x Y co x <4 ©' r4 co x ao © ci © © co Cl ©‘ © -4 -4	-4
C" _______Ct — Cl Cl ©©Cl ©©©©CO© ©©©©UP©^©© © '-O © © © ©_________________________________________________©
<	2. 03	•>•	nJ	n' •	•	...
O = g =3	K 02 aia5^> 02	02 02 02	02 (S 02 "Z2 02 02 X 02
* Q f ..	....................... ...	•	..........................
©CIOOO « - © — — © © Ci — O — Cl © © Cl © Cl Cl Cl — 71 Cl Cl
gpno[3JOJ lUvjnO — OOOOOC'©© 3 0 OOOOOOOOOOSOOOO©
i	...- ■■—----------- — -	- 2
i	©x	©©	©	x . .	..ci—;,	.	©
t4 UIOJB0 JO U[	en-j <ci x <S ci ci© © ai ©2 © ci © © x ©. ©	©©ci©©©	©	©
j	I	*>? “M CJ	'N V? CQ *?>	C1? O	Ol Of ,7f O? GQ
<	-------------------E	3	ro	o Jj	<-
*■“’ o	o	»J
•uiBHJ°*uI	o	co	co	&	g:
—-------^HfT - ci © -c © © r^x s-. ©^ ci © t; ©ig ^x © © -	?,	© ~r
*Hazy.
				

